# Analisys of pectoralis major tendon in weightlifting athletes using ultrasonography and elastography

**DOI:** 10.1590/S1679-45082015AO3335

**Published:** 2015

**Authors:** Alberto de Castro Pochini, Mario Ferretti, Eduardo Felipe Kin Ito Kawakami, Artur da Rocha Corrêa Fernandes, Andre Fukunishi Yamada, Gabriela Clemente de Oliveira, Moisés Cohen, Carlos Vicente Andreoli, Benno Ejnisman

**Affiliations:** 1 Escola Paulista de Medicina, Universidade Federal de São Paulo, São Paulo, SP, Brazil.; 2 Hospital Israelita Albert Einstein, São Paulo, SP, Brazil.

**Keywords:** Tendinopathy/ultrasonography, Pectoralis muscles/ultrasonography, Muscles/injuries, Elasticity imaging techniques

## Abstract

**Objective:**

To evaluate tendinopathy of the pectoralis major muscle in weightlifting athletes using ultrasound and elastography.

**Methods:**

This study included 20 patients, 10 with rupture of the pectoralis major muscle and 10 control patients. We evaluated pectoralis major muscle contralateral tendon with ultrasonographic and elastography examinations. The ultrasonographic examinations were performed using a high-resolution B mode ultrasound device. The elastography evaluation was classified into three patterns: (A), if stiff (more than 50% area with blue staining); (B), if intermediate (more than 50% green); and (C), if softened (more than 50% red).

**Results:**

Patients’ mean age was 33±5.3 years. The presence of tendinous injury measured by ultrasound had a significant different (p=0.0055), because 80% of cases had tendinous injury *versus* 10% in the Control Group. No significant differences were seen between groups related with change in elastography (p=0.1409).

**Conclusion:**

Long-term bodybuilders had ultrasound image with more tendinous injury than those in Control Group. There was no statistical significance regarding change in tendon elasticity compared with Control Group.

## INTRODUCTION

To gain strength for competitive sports activities and in weight training both for aesthetic reasons or improve quality of life have led a number of people to gyms and to participate in weight-lifting exercises. Brazil gym industry is the second largest in the world. Bench press exercise is the most popular one to strengthen upper limbs. A variety of national and international competitions has this exercise, which is standard exercise for paralympic weight-lifting. The pectoralis major muscle (PMM) injury has increased in last years for excessive weight-bearing, use of anabolic steroids, and wrong execution of bench press exercise.^(1-3)^ Direct injuries (trauma and contusions) occur in contact sports such as rugby, American football and skateboard.^(3-5)^

Pectoralis major muscle can be divided into two portions: clavicular which originates in external medial clavicle, and sternocostal which originates in sternum, body of sternum, and cartilages of the first to seven ribs. Injuries can be classified as total or partial rupture depending on percentage of fibers involved.^(6-8)^

Identifying morphologic changes or elastic properties of soft tissues with imaging methods has become an important and growing focus of research in order to obtain a more precisely assessment of injury and stiff tissues. Among these methods, the elastography is emphasized, which can be performed by high-resolution ultrasound (US) or magnetic resonance imaging. Because US elastrography or sonoelastography is easier to do and has a lower cost, they have been used in large scale, especially in mastology and liver diseases in which the use is employed in cases of differentiation among solid or cystic injuries and even for malignant and benign injuries, which can avoid invasive procedures such as biopsy.^(9-12)^

In high-resolution B mode US occurs a transformation of ultrasound waves reflected from moving object in audio sign or colors, being the two-dimensional analysis of target structure.^(13-15)^

Elastography seeks to analyze mechanical proprieties of viscosity and elasticity of medium sing compressive maneuvers, simulating the palpation method, used to evaluate the stiffness of a tissue. Images are acquired using emission and reception of short waves (US) and represent the internal deformity rate of tissues when they are submitted to mechanical tension. If a tissue has different stiffness of the others, then its deformity can be wider or narrow than the remaining medium evaluated. The more stiff the tissue, the little its deformation.^(16,17)^

There is variety of ways to evaluate tissue elasticity (“stiffness”) using US in which the last analysis depends on the type of excitation applied and how the tissue displacement is registered or measured.

Compression elastography is based on comparison of waves emitted before and after tissue compression that can be internal (heart beats) or external (manual compression). This technique has disadvantages because of its incapability to obtain quantitative data and for being an operating system dependent.^(13)^

In the acoustic radiation force impulse imaging (ARFI), the source of tissue excitation is not mechanical. Ultrasound pulses are generated by the device and focused on a specific area. In this case, the qualitative and quantitative assessment are possible, with reduction of operating system dependency factor.^(14)^

There is also supersonic shear imaging that main physical principle is similar to the ARFI, however the studied area is wider, with excitation pulses transmitted in a number of depths. As a disadvantage, there is depth limitation of tissues analysis.^(13)^

Assessment of chronic degeneration PMM tendon during weight bearing activities due to training, associated or not with prohibit substances before an injury, is limited by US technique and conventional magnetic resonance. New techniques development such as elastography is expected to obtain more information regarding integrity and degree of modification of tendon structure due to weight bearing activities in such way that factors enable the early diagnosis of degenerative changes in PMM tendon. Therefore, the adequate and early guidance would avoid a possible tendon rupture as well as difficulties in treatment, higher cost and uncertainty about the practitioner or athlete’s level of recovery.

## OBJECTIVE

To evaluate pectoralis major muscle rupture in weightlifting athletes using the ultrasonography and elastography.

## METHODS

We included 20 male weightlifting athletes, aged between 30 and 40 years old. Of these, 10 had history of PMM tendon injury opposed to the evaluated site within a period of 1 year after PMM rupture and 10 were control patients. We excluded those who were unable to perform the exam or missed follow-up consultations. All participants signed the consent form before the assessment. This study followed the ethical principles stated by resolution number 196/96 of the National Health Council. This project was approved by the Ethical and Research Committee of the *Universidade Federal de São Paulo* (UNIFESP) under the number 390.249 and CAAE: 20959813.0.0000.5505.

Ultrasound exams were performed at the clinic of the discipline of Sport Medicine in the Department of Ortopedics and Traumatology of the School of Medicine of UNIFESP, using a high-resolution US device (LOGIC P6, GE Healthcare) with 7 to 11MHz linear transducer.

All patients performed the B mode elastrography assessment. Exams were carried out with the patient in supine position, and arm in neutral position. Acquired images were evaluated by two radiologists with 4 and 11 years of experience on osteoarticular US, without access to clinical data and even access to patients’ physical exams.

**Figure 1:**
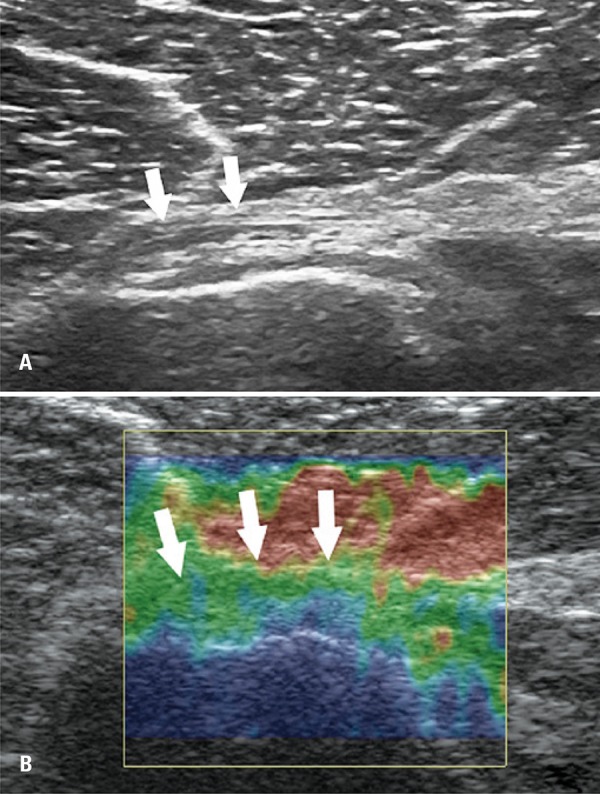
Ultrasound assessment of normal pectoralis major muscle tendon. (A) Normal tendon with fibrilar pattern and common echogenicity (white arrows). (B) Elastography assessment showing intermediate pattern (Green)

**Figure 2:**
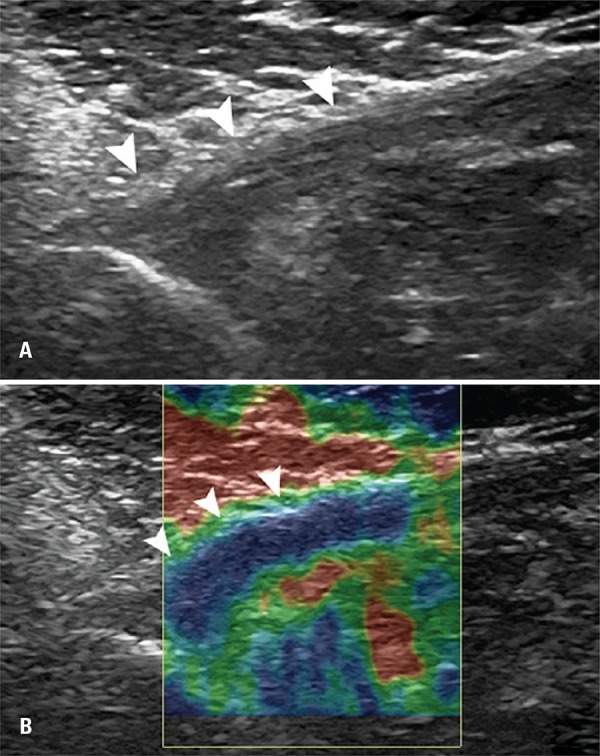
Ultrasound assessment of pectoralis major muscle tendon with signs of tendinous injury. (A) Thickened tendon and reduced ecogenicity – tendinous injury (arrowheads). (B) Elastography assessment showing stiff pattern (blue)

In the B mode analysis, we evaluate integrity, thickness, echogenicity and entheseal abnormalities. The measurement of PMM tendon thickness was performed immediately after the insertion in humeral diaphysis – in its medial segment. Changes of PMM were graded in normal tendon (homogeneous echotexture and fibrillar pattern), tendinous injury (thickness keeping the fibrillar pattern, reduction of echogenicity with or without thickeness) (Figures 1 and 2).

Elastography assessment was carried out applying repetitive and uniformity compressions with transducer with topography of PMM tendon insertion. The uniformity of compressions was standardized by graphic control showed simultaneously on a screen during the exam. The elastography was classified in three patterns: (A), if stiff (more than 50% of blue-stained area); (B) if intermediate (more than 50% of the green-stained area), and (C) if softened (more than 50% of red-stained area) (Figures 1 and 2).

## RESULTS

Participant’s mean age was 33±5.3 years. All 20 patients underwent assessment for change in PMM tendon opposite to the injury (progressive rupture) by two radiologists who had 4 and 11 years of experience in musculoskeletal area. No patients were lost during follow-up for US and elatography (Figures 1 and 2).

Tendinous injury in US findings between groups (p=0.0055) were statistically significant (Table 1). The percentage of athletes with tendinous injury in Case Group (80%) was significantly higher than in Control Group (10%). Changes in elastography did not show statistical significance between groups (p=0.1409).

**Table 1:** Assessment of athletes in ultrasound and elastography according to group (case or control)

Assessments	Case Group (n=10)	Control Group (n=10)	Total (n=20)	p value*
Ultrasonography: tendinous injury, n (%)				
No	2 (20)	9 (90)	11 (55)	0.0055
Yes	8 (80)	1 (10)	9 (45)	
Elastography, n (%)				
Blue	5 (50)	0 (0)	5 (25)	
Green	5 (50)	9 (90)	14 (70)	
Red	0 (0)	1 (10)	1 (5)	
Elastography: change, n (%)				
Normal (green)	5 (50)	9 (90)	14 (70)	0.1409
Change (blue or red)	5 (50)	1 (10)	6 (30)	

*p value Fisher’s exact test.

## DISCUSSION

The use of elastography to assess tendons constitutes a new and promising method in the orthopedic and sport medicine.^(18)^

Currently, most of orthopedists, when facing tendon or muscles injuries in athletes, have used magnetic resonance as the major exam.^(19-21)^ There are recent studies that show the importance of US and, more recently, the elastography to assess these tissues, which represent a decrease of costs to diagnose musculotendinous injuries.

The US is a well-established method to diagnose soft tissues injuries.^(5,22,23)^ Elastography is still a recent method in orthopedics and sport medicine. This method is most used in gynecology to evaluate breast injuries when the goal is to assess tissue stiffening, and it helps to avoid, in some cases, the need of local puncture and invasive procedures. Elastography, which is based on tissue response to compression maneuvers, can evaluate tissue qualitatively (blue and red) and quantitatively (new devices with more resources). The device we used in our patients performed a qualitative tissue assessment by tissue staining. The blue staining represents a stiffer tissue (less elastic) which would be associated to collagen degeneration (collagen type I metaplasia for the type III and angiofibroblastic proliferation). We did not find statistically significance different in relation to PMM tendon between those who practice weight-lifting and the Control Group. The result found may be influenced the criteria adopted by radiologists to analyze images, especially because there are well-established protocols for qualitative assessment of tendons. In case of weight-lifting athletes or weight-lifting practitioners, the current imaging protocols are unable to characterize in exams (US or magnetic resonance) the tendinous injury degree, such as the characterization of rotator cuff tendons (about 50% of degenerative change of tendon thickness). Acquisition protocols lack this type of images.

One of this study objectives was to evaluate tendons with smaller or greater change than 50% in terms of change in staining, thickness, and change in echogenic fibers in B mode US.

In this case, the US was an important tool to evaluate tendons of these athletes, and it also showed statistical difference between the two groups.

The importance of this assessment by imaging in this injury makes an analogy to calcaneus or patellar rupture that need urgent surgical treatment for reinsertion. The majority of athletes or weight-lifting practitioners with PMM rupture does not count with preventive assessment of this tendon, such as in rotator cuff and after injury. Most of injuries is not diagnosed in emergency room because it is more linked with sport medicine than traditional orthopedics. The consequence of this diagnostic problem is that patients seek treatment when they are in chronic stage (after 1 month of injury) and with significant loss in isokinetic assessment that can range from 25% to 50% in adduction strength of the shoulder,^(24-27)^ which represent a significant loss in performance of athletes who sport involves the upper limbs. In these cases, tendon reconstruction is need (using autologous tendon graft) instead of the repair (which can be done in injuries with less than 3 weeks), causing, therefore, large morbidity to the chronic procedure. The reconstruction of PMM tendon is commonly performed using other tendons, because after total rupture, which is commonly seen in chronic stages, there is no tendon stumps close to the humerus. The most used technique is based on use of semitendinosus and gracilis tendon of the knee, which gives the patient a more aggressive treatment when compared to repair acute tendon.^(5,8,28-30)^

The detection of degenerative change of PMM tendon in earlier phases, using US or elastography can help to guide patients and also the professionals surrounding them, such as physical educators and physical therapists. To establish the risk of rupture based on images is impossible, both for rotator cuff of the shoulder and for patella ligament or calcaneal tendon. However, guidance of changing degree (more or less than 50) can help to perceive need to change training and relative overload to these tendon, especially the PMM.

Results did not show significant differences between the Case Group and Control Group.

Elastography did not show statistically significant difference, however, in the Control Group, no patients had blue staining in tendons (stiffening) and there was a case with assessment that staining was red (intermediary stiffness). But in the Case Group (athletes) we observed five cases of imaging assessment of tendon with blue staining (stiff) and five cases with green staining in the PMM tendon (less stiff). Some factors may influence this result, such as the little experience of the radiologist with the technique because it is a new approach which does not have specific established protocols. Elastography is a promising method in functional assessment of musculotendinous tissues. New protocols and techniques for imaging acquisition are need, and further studies must be carried out in order to clarify the real role of this new technique in orthopedics and sport medicine.

The number of patients (Case Group) included in our study was a limitation, but PMM tendon rupture is still an infrequent injury, and this injury early or chronic diagnosis is uncommon. Before this study, our group published a study that included the largest sample of PMM ruptures in athletes reported in literature.^(8)^ The study followed-up 60 patients, but most of them had injuries for more than 1 and, therefore, they were not no elective for the present study.

## CONCLUSION

Long-term weight-lifting practitioners showed in ultrasonography images higher incidence of tendinous injury compared with Control Group. Despite the quality of elastography and its sensible to show changes in stiffness of athletes tendons, this procedure did not show statistically significance in elastic changes in tendons when the two groups were compared.
