# Supplementary material for: Analisys of pectoralis major tendon in weightlifting athletes using ultrasonography and elastography
Source: Einstein (Sao Paulo). 2015 Oct-Dec;13(4):541–6. doi: 10.1590/S1679-45082015AO3335 (PMC4878628; doi:10.1590/S1679-45082015AO3335)
Supplement: Translation

# Translation: Estudo do tendão do músculo peitoral maior utilizando ultrassonografia e elastografia em atletas de levantamento de peso

**Authors:** Alberto de Castro Pochini, Mario Ferretti, Eduardo Felipe Kin Ito Kawakami, Artur da Rocha Corrêa Fernandes, Andre Fukunishi Yamada, Gabriela Clemente de Oliveira, Moisés Cohen, Carlos Vicente Andreoli, Benno Ejnisman

**Affiliations:** 1 Escola Paulista de Medicina, Universidade Federal de São Paulo, São Paulo, SP, Brasil.; 2 Hospital Israelita Albert Einstein, São Paulo, SP, Brasil.

**Keywords:** Tendinopatia/ultrassonografia, Músculos peitorais/ultrassonografia, Músculos/lesões, Técnicas de imagem por elasticidade

## Abstract

**Objetivo:**

Avaliar tendinopatia do músculo peitoral maior em praticantes de levantamento de peso utilizando ultrassonografia e elastografia.

**Métodos:**

Participaram do estudo 20 sujeitos, sendo 10 com ruptura do tendão do músculo peitoral maior e 10 como controles. Avaliou-se o tendão músculo peitoral maior contralateral por meio de exames ultrassonográficos e elastografia. O aparelho de ultrassonografia utilizado era de alta resolução, e a avaliação foi realizada no modo B. A avaliação por elastografia foi classificada em três padrões, a saber: (A) se endurecido (mais de 50% de área com coloração azul); (B), se intermediário (mais de 50% verde); e (C), se amolecido (mais de 50% vermelho).

**Resultados:**

A média de idade da amostra foi 33±5,3 anos. Foi encontrada diferença estatisticamente significante (p=0,0055) quanto à presença de tendinopatia avaliada pela ultrassonografia, pois 80% dos casos apresentaram tendinopatia músculo peitoral maior *versus* 10% nos pacientes controles. Não foram encontradas diferenças significantes entre os grupos quanto à presença de alteração na elastografia (p=0,1409).

**Conclusão:**

Os pacientes praticantes de musculação de longa data apresentaram imagem à ultrassonografia com maior tendinopatia em relação aos controles, e não foi obtida significância estatística quanto à elastografia em relação aos controles.

## INTRODUÇÃO

A busca pela força nas atividades esportivas competitivas e na musculação, seja por motivos estéticos ou por qualidade de vida, tem levado muitas pessoas às academias de ginástica e ao treino de força. Estima-se que o Brasil seja o segundo país do mundo em número de academias. Um dos exercícios mais realizados em academias para o fortalecimento do membro superior é o supino. Várias competições nacionais e internacionais envolvem esse exercício, que se trata de um exercício padrão do levantamento de peso paraolímpico. A lesão do músculo peitoral maior (MPM) tem aumentado nos últimos anos por excesso de carga, uso de esteroides anabolizantes e execução de movimentos incorretos na prática do treino de força no exercício de supino.^(1-3)^ As lesões diretas (trauma e contusões) ocorrem em esportes de contato como *rugby*, futebol americano e *skate*.^(3-5)^

O MPM pode ser dividido em duas porções: clavicular, com origem na metade external da clavícula, e esternocostal, com origem no manúbrio, corpo do esterno e cartilagens costais (primeira a sétima). As lesões podem ser classificadas em rupturas totais ou parciais, de acordo com a porcentagem de fibras envolvidas.^(6-8)^

A identificação das alterações morfológicas ou de propriedades elásticas dos tecidos moles com o uso dos métodos de imagem tem se tornado um importante e crescente foco de pesquisa, visando obter uma avaliação mais precisa do grau de dano e do enrijecimento dos tecidos. Entre esses métodos, destaca-se a avaliação por elastografia, que pode ser realizada com o uso de aparelhos de ultrassonografia (US) ou ressonância magnética. Por ser de mais fácil acesso e com menor custo, a elastografia por US ou sonoelastografia vem sendo utilizada em maior escala, especialmente nas áreas de mastologia e hepatologia, nas quais seu uso é empregado nos casos de diferenciação entre lesões sólidas ou císticas e até mesmo entre lesões malignas e benignas, podendo evitar procedimentos invasivos como uma biópsia.^(9-12)^

Na US modo B, ocorre a transformação das ondas de ultrassom refletidas de um objeto em movimento em um sinal de áudio ou cores, sendo possível a análise bidimensional da estrutura alvo.^(13-15)^

A elastografia busca analisar as propriedades mecânicas de viscosidade e elasticidade de um meio, por manobras de compressão, simulando o método de palpação, utilizado para avaliar a rigidez de um tecido. As imagens são adquiridas por meio da emissão e recepção de ondas curtas (US) e representam a taxa de deformação interna dos tecidos, quando estes são submetidos a uma tensão mecânica. Se algum tecido possuir rigidez diferente dos demais, então sua deformidade pode ser maior ou menor que o restante do meio avaliado. Se for mais rígido, sua deformação é menor.^(16,17)^

Existem várias maneiras de avaliar a elasticidade (“dureza”) tecidual pela US, a qual, em última análise, depende do tipo de excitação aplicada e de como o deslocamento tecidual é registrado ou medido.

A elastografia por compressão baseia-se na comparação das ondas emitidas antes e depois de uma compressão tecidual, podendo ser interna (batimentos cardíacos) ou externa (por meio de compresso manual). Esta técnica tem como desvantagem a incapacidade de obter dados quantitativos e por ser examinador-dependente.^(13)^

Na técnica de força de radiação acústica (ARFI - *acoustic radiation force impulse imaging*), a fonte de excitação tecidual não é mecânica. Pulsos de ultrassom são gerados pelo aparelho e focados em área específica. Neste caso, a avaliação qualitativa e quantitativa são possíveis, com redução do fator operador-dependente.^(14)^

Há também a imagem de cisalhamento supersônico, cujo princípio físico é semelhante ao do ARFI, porém a área estudada é maior, com pulsos de excitação transmitidos em várias profundidades. Como desvantagem, há uma limitação na profundidade de análise dos tecidos.^(13)^

A avaliação da degeneração crônica do tendão MPM por sobrecarga vindo do treinamento, associado ou não ao uso de substâncias proibidas, antes de uma eventual lesão, é limitada pelos métodos de US e ressonância magnética convencionais. Com o desenvolvimento de novas técnicas, como a elastografia, espera-se obter mais informações quanto à integridade e ao grau de modificação estrutural do tendão, devido à sobrecarga, de modo que tais fatores permitam o diagnóstico precoce de alterações degenerativas no tendão do MPM. Desta forma, a orientação adequada e precoce evitaria uma possível ruptura tendínea, bem como maior dificuldade do tratamento, maior ônus e a incerteza do grau de recuperação do praticante ou atleta.

## OBJETIVO

Avaliar tendinopatia do músculo peitoral maior em praticantes de levantamento de peso utilizando ultrassonografia e elastografia.

## MÉTODOS

Foram avaliados incluídos 20 atletas do sexo masculino praticantes de levantamento de peso, entre 30 e 40 anos, sendo 10 com histórico de lesão do tendão do MPM contralateral ao lado avaliado dentro do período de 1 ano da ruptura MPM e 10 controles. Foram excluídos do estudo pacientes com impossibilidade de realizar o exame ou perda de acompanhamento. Todos assinaram o Termo de Consentimento Livre e Esclarecido antes da avaliação, de acordo com a Resolução 196/96, do Conselho Nacional de Saúde, aprovado pelo Comitê de Ética em Pesquisa da Universidade Federal de São Paulo (UNIFESP), parecer número 390.249 e CAAE: 20959813.0.0000.5505.

Os exames ultrassonográficos foram realizados no ambulatório da Disciplina de Medicina Esportiva, no Departamento de Ortopedia e Traumatologia da Escola Paulista de Medicina da UNIFESP, utilizando aparelho de US de alta resolução (LOGIC P6, GE Healthcare), com transdutor linear de 7 a 11MHz.

Em todos os pacientes, foi realizada avaliação no modo B e com elastografia. Os exames foram realizados com o paciente em posição supina, com o braço em posição neutra. As imagens adquiridas foram avaliadas em consenso por dois radiologistas com 4 e 11 anos de experiência na área de US osteoarticular, sem acesso aos dados clínicos e nem aos exame físico dos pacientes.

Na análise no modo B, foram avaliados integridade, espessura, ecogenicidade e anormalidades da êntese. A medida da espessura do tendão do peitoral maior foi realizada imediatamente após a inserção na diáfise umeral, em seu segmento médio. As alterações do tendão do peitoral maior foram graduadas em tendão normal (ecotextura homogênea e padrão fibrilar), tendinopatia (espessamento mantendo padrão fibrilar, redução da ecogenicidade com ou sem espessamento) (Figuras 1 e 2).

**Figura 1:** Avaliação ultrassonográfica de um tendão do peitoral maior normal. (A) Tendão normal apresentando padrão fibrilar e ecogenicidade habitual (setas brancas). (B) Avaliação por elastografia demonstrando padrão intermediário (verde)

**Figura 2:** Avaliação ultrassonográfica de um tendão do peitoral maior com sinais de tendinopatia. (A) Tendão espessado e com ecogenicidade reduzida − tendinopatia (cabeça de setas). (B) Avaliação por elastografia demonstrando padrão endurecido (azul)

A avaliação por elastografia foi realizada aplicando compressões repetitivas e uniformes com o transdutor junto à topografia de inserção do tendão MPM. A uniformidade das compressões foi padronizada por controle de gráfico exibido simultaneamente à realização do exame na tela do aparelho. A elastografia foi classificada em três padrões: (A), se endurecido (mais de 50% de área com coloração azul); (B), se intermediário (mais de 50% de área com coloração verde); e (C), se amolecido (mais de 50% de área com coloração vermelho) (Figuras 1 e 2).

## RESULTADOS

A média de idade da amostra foi 33±5,3 anos. Em todos os 20 pacientes as imagens adquiridas foram avaliadas em consenso por dois radiologistas com 4 e 11 anos de experiência na área de ultrassonografia do sistema musculoesquelético buscando alterações no tendão do MPM contralateral a lesão (ruptura pregressa). Não houve perda de acompanhamento entre US e elastografia (Figuras 1 e 2).

Foi encontrada diferença estatisticamente significante entre os grupos quanto à presença de tendinopatia avaliada pelo US (p=0,0055) (Tabela 1). O porcentual de atletas com tendinopatia no Grupo Caso (80%) foi significantemente maior do que nos pacientes controles (10%). Não foi encontrada diferença estatisticamente significante entre os grupos quanto à presença de alteração na elastografia (p=0,1409).

**Tabela 1:** Avaliações dos atletas com relação à ultrassonografia e à elastografia, segundo o grupo de estudo (caso ou controle)

Avaliações	Grupo Caso (n=10)	Grupo Controle (n=10)	Total (n=20)	Valor de p*
Ultrassonografia: tendinopatia, n (%)				
Não	2 (20)	9 (90)	11 (55)	0,0055
Sim	8 (80)	1 (10)	9 (45)	
Elastografia, n (%)				
Azul	5 (50)	0 (0)	5 (25)	
Verde	5 (50)	9 (90)	14 (70)	
Vermelho	0 (0)	1 (10)	1 (5)	
Elastografia: alteração, n (%)				
Normal (verde)	5 (50)	9 (90)	14 (70)	0,1409
Alterado (azul ou vermelho)	5 (50)	1 (10)	6 (30)	

* Valor de p teste exato de Fisher.

## DISCUSSÃO

O uso da elastografia para avaliação dos tendões representa um método novo e promissor dentro da ortopedia e da medicina esportiva.^(18)^

A maior parte dos ortopedistas, diante de lesões tendíneas ou musculares em atletas competitivos, atualmente tem buscado a ressonância magnética como principal exame.^(19-21)^ Há estudos recentes que mostram a importância da US e, mais recentemente, da elastografia na avaliação destes tecidos, o que representaria uma diminuição dos custos envolvendo diagnóstico de lesões musculotendíneas.

A US representa método já consagrado na prática diagnóstica de lesões de tecidos moles.^(5,22,23)^ A elastografia ainda é método recente dentro da ortopedia e da medicina esportiva, sendo mais utilizada em especialidades como ginecologia, para avaliação de lesões de mama, quando se deseja avaliar o endurecimento do tecido, evitando, em alguns casos, até a necessidade de punção local e procedimento invasivo. A elastografia, que se baseia na resposta do tecido a manobras de compressão, pode avaliar o tecido com informações qualitativa (cores de azul a vermelho) e quantitativa (aparelhos mais recentes com mais recursos). O aparelho utilizado nos nossos pacientes realizava avaliação qualitativa do tecido por meio da coloração do tecido. A coloração azul representava tecido mais rígido (menos elástico), o que estaria mais associado à degeneração do colágeno (metaplasia de colágeno tipo I para tipo III e proliferação angiofibroblástica). Não foi encontrada diferença estatística entre os grupos de atletas praticantes de musculação e controles quanto ao tendão do MPM. O resultado também pode ter sido influenciado pelo critério do radiologista em analisar as imagens, já que ainda não há protocolos bem estabelecidos para avaliação qualitativa dos tendões. No caso de atletas de levantamento de peso ou musculação com os protocolos atuais de imagem, não é possível caracterizar por exames (US ou ressonância magnética) o grau de tendinopatia, como fazemos referente aos tendões do manguito rotador do ombro (mais ou menos de 50% de modificação degenerativa da espessura do tendão). Faltam protocolos de aquisição de imagens para esse fim.

Um dos objetivos deste trabalho foi propor a avaliação dos tendões em maior ou menor que 50% de modificação da coloração, e espessamento e alteração de ecogenicidade das fibras à US no modo B.

Neste caso, a US se mostrou importante ferramenta em avaliar os tendões desses atletas, e foi observada diferença estatística entre os dois grupos de pacientes.

A importância dessa avaliação por imagem nessa lesão faz analogia à ruptura do tendão calcâneo ou patelar, que necessitam do tratamento cirúrgico de urgência para reinserção. A maior parte dos atletas ou praticantes de musculação com ruptura do MPM não contam com avaliação preventiva desse tendão, como no manguito rotador e após a lesão. A maior parte não é diagnosticadas no pronto-socorro, por se tratar de lesão mais relativa ao estudo da medicina esportiva que da ortopedia tradicional. A consequência deste problema diagnostico é a busca pelo tratamento dos pacientes nos estágios crônicos (após 1 mês de lesão) e com perda de força na avaliação isocinética, que pode variar de 25 a 50% na força de adução do ombro,^(24-27)^ o que representa importante perda do desempenho do atleta que utiliza do membro superior. Nesses casos, é necessária a reconstrução do tendão (utilização de enxerto de tendão autólogo), em vez do reparo (que pode ser realizado em lesões com menos de 3 semanas), agregando, assim, maior morbidade ao procedimento crônico. A reconstrução do tendão MPM habitualmente é realizada utilizando outros tendões, já que, após a ruptura total, observada comumente nos estágios crônicos, não restam cotos de tendão junto ao úmero. A técnica mais utilizada é baseada no uso dos tendões semitendíneo e grácil do joelho, o que confere ao paciente um tratamento mais agressivo quando comparado ao reparo do tendão em fase mais aguda.^(5,8,28-30)^

Assim a detecção de modificação degenerativa do tendão MPM em fases mais precoces, utilizando US ou elastografia, pode auxiliar na orientação dos pacientes e profissionais que o cercam, como educadores físicos e fisioterapeutas. Não é possível estabelecer risco de ruptura baseado nas imagens, tanto para o manguito rotador do ombro, como para o ligamento patelar ou tendão calcâneo. No entanto, a orientação do grau de modificação (maior ou menor que 50%) pode ajudar a percepção da necessidade em modificar o treino e sobrecarga relativa a esses tendões, em especial ao MPM.

Os resultados não apontaram diferenças significativas entre os Grupo Caso e Grupo Controle.

A elastografia não apresentou diferença estatisticamente significante, entretanto, no Grupo Controle, nenhum paciente apresentou coloração azul nos tendões (enrijecimento) e houve um caso com avaliação cuja coloração foi vermelha (enrijecimento intermediária). Já no Grupo Caso (atletas), foram encontrados cinco casos de avaliação da imagem do tendão com coloração azul (rígido) e cinco casos com coloração verde no tendão MPM (menos rígido). Alguns fatores podem ter influenciado neste resultado, como a falta de uma maior familiarização do radiologista com o método e a técnica, pois se trata de método recente, ainda sem protocolo específico. A elastografia representa método promissor na avaliação funcional dos tecidos musculotendíneos. Novos protocolos e técnicas de aquisição de imagens são necessários, e futuros estudos deve elucidar o real papel desse novo método na ortopedia e na medicina esportiva.

O número de pacientes (Grupo Caso) utilizado nesta pesquisa representou uma limitação no desenho do estudo, mas a ruptura do tendão do MPM ainda representa lesão não tão frequente e cujo diagnóstico precoce ou crônico não é comum. Assim, nosso grupo publicou anteriormente a maior casuística da literatura mundial do caso^(8)^ com 60 pacientes em acompanhamento, mas boa parte deles com lesões acima de 1 ano e, portanto, não eletivos ao estudo em questão.

## CONCLUSÃO

Os pacientes praticantes de musculação de longa data apresentaram imagem à ultrassonografia com maior tendinopatia em relação aos Grupo Controle e, embora a elastografia qualitativa se mostrasse sensível às modificações de rigidez aos tendões dos atletas, não foi obtida significância estatística quanto à modificação elástica dos tendões em relação aos controles.
